# The Hospital

**Published:** 1918-02-16

**Authors:** 


THE
### INSTITUTIONAL WORKER
Being a Special Supplement to "The Hospital"

### OUR BUREAU OF INFORMATION.

### Rules for Correspondents.

1. Every letter must be accompanied by the coupon to be cut from the back cover (inside page) of The Hospital, current issue, and must contain the name and address of the correspondent with pseudonym for publication if desired. Replies by post cannot be given save under exceptional circumstances at the Editor's discretion.

2. Letters from Approved Homes in reply to special needs published in the Bureau should state terms and full particulars, and be sent prepaid under cover to the Editor of the Bureau with name written across coupon for identification.

3. Proprietors of Homes which have not yet been entered on the List of Approved Homes, but have spare accommodation likely to suit special needs, are invited to write for an application form for registration. The fee for registration, which includes two announcements of the Home in the Bureau and other privileges, is 10s.

4. All communications to be addressed to the Editor of The Hospital, 28 Southampton Street, Strand, London, W.C. 2, and marked "Bureau of Information."

### INSTITUTION FACTS AND FIGURES.

### Extending a Hospital Engineer's Work.

#### Answer to January Question.

By M. K. S.

The question was:

**In what ways can a hospital engineer be useful in addition to his routine work of attending to the heating apparatus?**

On the outbreak of war in August 1914 one of the most urgent and difficult problems to face in a great many institutions was the calling-up of the engineer, who, in nineteen cases out of twenty, was an old Navy rating Reserve or Army ditto.

The all-round handyman produced by the training of the Royal Navy was indeed a treasure, and the easy working of the hospital largely depended on his efficiency and the cheerful way he had of carrying out orders.

His duties consisted primarily of running the plant of the steam laundry and heating apparatus of the hospital, nurses' home, etc. He superintended the cleaning of all boilers, the running of the washing machines and hydros in the laundry, the stoking of all furnaces, the minor repairs of the plant of same, refixing felt on calendar, etc., and the cleaning of the wash-house itself. He was also responsible for the burning of all surgical waste, the disinfecting of mattresses and bedding, and in many cases the sterilising of the surgical-dressing drums other than those attended to in the theatre. The minor electrical and plumbing repairs also came under his supervision—*i.e.*, the fixing of new washers, plunging sinks, repairing minor leakages, etc.

Once weekly he also checked and entered the gas and water meters, and where the water was hard, and boiler fluid had regularly to be added to the cistern, this also came in the day's work.

With all these numerous duties the engineer must necessarily be a very competent and efficient workman, and since the war called up its reserves, hospitals have suffered exceedingly from substitutes of all kinds. The staff under the engineer, of course, varies with the size of the institution—usually a stoker and a boy. He would also require assistance with the stacking of steam-coal, riddlings, etc., when there was a pressure of urgent work—as after a severe frost.

There are many incidental duties which would also devolve on the engineer, such as seeing that cinders are sifted and used again, and that the gutters and drains are regularly flushed, and the collection of grease from the different wash-up sinks is removed. The "blowing" of gas-rings and cleaning of ovens very often also falls to his work.

### Crockery Washing in a Nurses' Home.

#### Answer to January Question.

By "Rita."

The question was:

**Describe the cleanest, quickest, and most careful method of washing and drying crockery in a nurses' home to accommodate 180 persons.**

A wooden tub is always preferable to any other for the washing of crockery. It saves many breakages and much chipping.

Glasses that have contained milk should be rinsed out with cold water first, and then be washed in clear warm water and dried on very clean dry cloths and polished.

Cups and saucers should be separated, and the saucers set in a pile at one end of the tub, with the cups in piles at the other end. After the tub has been filled with hot water, to which a little soda has been added, the saucers should be washed first, and as they are done should be dipped into a tub of clean cold water and drained on a perforated board. The cups can then be treated likewise, and by the time the cups are washed and rinsed the saucers will be found to be nearly dry, and will need very little finishing on a dry cloth. The cups can then be finished in the same way. While finishing the cups the plates can be put into the tub to soak. They are washed and rinsed in the same way, but instead of being drained on the perforated board should be put into a plate-rack to dry, and left there until needed again, when they will require a final polish.

Forks and spoons should be washed in hot soapy water, rinsed in hot water, and dried at once.

Knives should be put into a jug or shallow bowl of soapy water up to the handle to soak while the other things are being done; they can then be washed after the spoons and forks.

Plunging the crockery into cold water after it has been washed in hot water helps it to dry very quickly, and is the cleanest and most expeditious way of dealing with it.

### FEBRUARY QUESTIONS.

### (1) Hot Dinner-Plates.

A hospital has four wards, each containing twenty beds. The dinners are served from one inadequate general kitchen and there is no arrangement for heating the plates. Each ward has a very tiny kitchen, which holds a small gas-ring. How can the plates be best heated?

### (2) How to Sterilise Rubber Gloves.

Describe the best method of sterilising rubber gloves, to be used during operations, to ensure their being perfectly sterilised with the minimum amount of damage to the rubber.

### RULES.

The following rules must be observed:—

1. Contributions must be written on one side of the paper. Brevity and terseness are desirable features. The MSS. must bear the name and address of the sender and be accompanied by coupon to be cut from the back cover (inside page) of the current issue of The Hospital. A pseudonym must be chosen if the name is not to be published.

2. Contributions must be addressed to the Editor of The Hospital, Institutional Worker Supplement, 28 & 29 Southampton Street, Strand, London, W.C. 2, should reach him before the end of the current month, and be marked in the left-hand corner "Facts and Figures."

**A minimum payment of Five Shillings will be made for each published answer.**

### QUESTIONS INVITED.

In connection with our Question Box, we offer every month five shillings each for the two best questions which are sent in for consideration. The questions may be on any institutional subject and concern any department, from the secretary's to the porter's, or the matron's to the domestic staff; the one essential is that they must be practical and deal with points that have a definite relation to hospital or institutional administration, upkeep, finance, or management. Points relating to artificers' work, housekeeping, the laundry, out-patients, and other practical matters will be welcome. It is hoped that thus, when difficult or doubtful points arise in the course of their work, institutional workers will be encouraged to put them in the form of a question and send them to the Editor, The Hospital Bureau of Information, 28 & 29 Southampton Street, Strand, W.C. 2, marked "Question Box."

The best questions will be published in due course and our readers will be given the opportunity of answering them. Thus all institutional workers may be able to cooperate with the view of helping the work and smoothing the difficulties of each other. In short, we want the Question Box of the *Institutional Worker* to be freely used by every worker habitually.

### ENQUIRIES AND ANSWERS.

### *WAR MATTERS.*

### Gifts of Walking-Sticks for the Wounded.

We suggest that you write to Queen Mary's Needlework Guild, St. James's Palace, S.W., in regard to the stout crook-handled walking-sticks for the use of the wounded in war hospitals.—E. M. W.

### *EMPLOYMENT AND TRAINING.*

### Training of Dispenser.

The highest degree a woman dispenser can aspire to is that of pharmaceutical chemist, but this necessitates a long course of study. You can qualify to work under a doctor only by passing the Assistants' Examination of the Society of Apothecaries of London, for which six or twelve months is sufficient time for study. Write to the Secretary, Pharmaceutical Society, 17 Bloomsbury Square, London, W.C., for any further particulars.—Tootle.

### Queen Victoria's Jubilee Institute for Nurses.

A three years' certificate from a general hospital or a Poor-Law infirmary recognised as a training-school is necessary before an application for district training can be considered by Queen Victoria's Jubilee Institute for Nurses. The six months' district training includes the necessary trial month.—Constance.

### Necessary Qualification for School Nurses.

The necessary qualifications of a school nurse are not strictly defined, but most authorities regard a three years' training at a recognised training-school (*i.e.*, a hospital of 100 beds or over) to be essential.—Kenny.

### *THE SICK AND IN NEED.*

### Home for Epileptic Woman.

We suggest that you apply to the following institutions on behalf of the woman suffering from epilepsy: The Home for Epileptics, Maghull, near Liverpool, and the Kingston St. Michael Home for Epilepsy, Chippenham, Wilts. The charges at both of these institutions are small. Epileptic women are also received at the Chalfont Colony, Bucks, and cases are admitted upon application to the National Society for Epileptics, Denison House, Vauxhall Bridge Road, S.W., for a small weekly payment.—Worried One.

### List of Sanatoria.

You will find a list of sanatoria in England in "Burdett's Hospitals and Charities," which can be seen at most public libraries; or if you enclose 7d. to the National Association for the Prevention of Consumption, 20 Hanover Square, London, they will forward you the list they issue.—Maurice N.

### *THE HOUSEKEEPERS' DEPARTMENT.*

### A War-time Dish.

You will probably find the following dish suitable for your requirements: Hard-boil the necessary number of eggs, remove the shells, and cut the eggs in halves; place on a dish and cover each half-egg with mashed potatoes. A little tomato sauce gives a piquancy to it.

### *MISCELLANEOUS.*

### Salvarsan Apparatus.

A good type of apparatus for the intravenous injection of salvarsan is supplied by Messrs. Evans Sons Lescher and Webb, Ltd., of 56 Hanover Street, Liverpool. Considerable pains have been taken in planning the apparatus to secure absolute sterility of the solution, and to prevent any possible irritation at the site of the injection. Clear and detailed directions for carrying out the operation are provided with each set.—House Physician.

### Travelling X-ray Department.

Your suggestion that certain small hospitals within a reasonable area should arrange among themselves for the provision of one portable *x*-ray equipment to supply the needs of all is quite feasible. By the adoption of this method a much more efficient service could be secured than would be possible if each hospital were to instal a small outfit for very occasional use, the efficiency of which must necessarily be limited. A good operator in charge of a powerful installation fitted in a motor-ambulance could undoubtedly do useful work for a number of small hospitals. Great strides have been made recently in fitting up ambulances of this character for foreign service. Messrs. Siemens Bros., Ltd., are specialising in this work with marked success.—Progressive.

### Stocks of Food.

We do not think that you as a hospital housekeeper need have any uneasy feeling in respect of your stocks of food during "Surrender Week" or after. In any case you will remember that a reasonable supply, say enough to last a fortnight, is allowed even to private people. From the list of quantities you send we should judge that with your 100 patients and 50 staff there is little to spare in respect of any one article. The amount of tinned meats (much of which you say has been given to the institution) appears at first sight to be a little heavy, but considering that you have for some weeks been unable to obtain more than three-fifths of your normal fresh meat supply we do not think that you have an unreasonable stock. You should not have more than enough sugar to last you until your next supply, so in respect of this article all depends upon when the next consignment is due.—Housekeeper.

